# Glutathione Peroxidases 1 and 3 Immunoscores in Clear Cell Renal Cell Carcinoma: New Insights from a Case-Series Study

**DOI:** 10.32604/or.2026.077195

**Published:** 2026-04-22

**Authors:** Dimitra P. Vageli, Panagiotis G. Doukas, Nikolaos Papageorgiou, Chrysanthi A. Markou, Konstantina Zacharouli, Maria Ioannou

**Affiliations:** 1 Department of Pathology, Faculty of Medicine, School of Health Sciences, University of Thessaly, Larissa, Greece; 2 Center of Neuroscience and Regeneration Research Center, Yale University School of Medicine & VA-Connecticut Healthcare System, West Haven, CT, USA; 3 Department of Neurology, Yale School of Medicine, New Haven, CT, USA; 4 Cancer Signaling Networks Program, Yale Cancer Center, Yale School of Medicine, New Haven, CT, USA; 5 Department of Medicine, Rutgers-Robert Wood Johnson Medical School/Saint Peter’s University Hospital, New Brunswick, NJ, USA; 6 Pathology Anatomy Laboratory, University General Hospital of Larissa, Mezourlo Larissa, Larissa, Greece

**Keywords:** GPX1, GPX3, clear cell renal cell carcinoma (ccRCC), immunohistochemistry, WHO/ISUP (nucleolar) grade

## Abstract

**Background:**

Renal cell carcinoma (RCC) is the most common type of kidney cancer in adults, with a poor prognosis in advanced stages. Although histological tumor grading is an established prognostic parameter, it often fails to capture the biological heterogeneity of RCC. Therefore, identifying novel biomarkers could enhance early diagnosis and improve predictive accuracy. Here, we aimed to test whether immunophenotypes of specific glutathione peroxidase (GPX) family members may have prognostic value in RCC.

**Methods:**

We investigated the relationship between GPX1 and GPX3 immunophenotypes and clinicopathological parameters in 32 surgical specimens of clear cell RCC (ccRCC) with nucleolar grade 1–4 (WHO/ISUP grading). We evaluated the GPX1 and GPX3 immunophenotypes and assigned a histological immunoscore for each marker. For analysis, we used Spearman and point-biserial correlation methods.

**Results:**

Our findings indicated a significant positive correlation between GPX1 immunoscore and high nucleolar grade (*r* = 0.80, *p* < 0.0001). In contrast, we observed a significant negative correlation between GPX3 immunoscore and high nucleolar grade (*r* = −0.77, *p* < 0.0001). We did not find statistically significant correlations between GPX1 expression and age, sex, tumor localization, or tumor size (*p* > 0.05), nor with capsular infiltration and invasion of the renal pelvis (*p* > 0.05). However, we did find statistically significant positive correlations between GPX1 expression and invasion of the renal vein (*p* = 0.038), perirenal fat (*p* = 0.043), and peripyelic fat (*p* = 0.015).

**Conclusion:**

Our data demonstrate that GPX1 and GPX3 immunophenotypes could have a prognostic role for ccRCC, particularly in relation to nucleolar grade. This study has limitations because of the small sample size; however, it underscores the necessity for further research in larger, prospective studies. These studies should more thoroughly examine the associations between GPX1 and GPX3 and clinicopathological parameters, and validate them as potential novel prognostic biomarkers.

## 1 Introduction

Renal cell carcinoma (RCC) is the predominant adult kidney malignancy, accounting for approximately 90% of renal cancers [1]. The global incidence of RCC is rising, with nearly 400,000 new cases reported annually [2]. RCC encompasses a range of histopathologic entities, and modern diagnosis relies on established morphologic criteria, along with immunohistochemistry and targeted molecular assays, following the 2022 World Health Organization classification [3]. Among the various subtypes of RCC, clear-cell RCC (ccRCC) is the most prevalent, representing around 70–80% of cases.

The etiology of RCC is multifactorial. The most well-established risk factors include tobacco smoking, excess body weight, hypertension, long-term hemodialysis with acquired cystic kidney disease, and family history/hereditary syndromes [4,5]. Certain occupational exposures and specific dietary habits may also significantly impact the risk of developing RCC [6,7]. Loss of chromosome *3p* and mutation of the von Hippel-Lindau (VHL) gene at *3p25* are commonly observed in ccRCC and are thought to promote tumor metastasis [8]. However, there is currently no evidence to support primary screening for RCC, except for certain individuals with a hereditary predisposition or a strong family history, for whom targeted genetic counseling and testing are recommended [9]. This highlights the importance of continuing to develop and validate biomarkers for RCC, which will aid in early diagnosis and prognosis.

The glutathione peroxidase (GPX) family is a vital component of the antioxidant system that metabolizes intracellular reactive oxygen species (ROS) and maintains cellular homeostasis [10,11], thereby protecting cell membranes from oxidative damage [12]. The GPX family consists of eight members (GPX1-8) in mammals, five of which are selenoproteins (GPX1-4 and 6) in humans, while the other three isoforms contain cysteine instead of selenocysteine [13].

The GPX1 gene maps to chromosome 3p21.3, a region often associated with loss of heterozygosity in RCC. The GPX1 protein is primarily found in the cytosol and mitochondria, where it catalyzes glutathione-dependent reduction of hydrogen peroxide and other hydroperoxides, thus supporting redox homeostasis [14]. GPX1 is particularly critical since it cannot be replaced by any other selenoprotein in protecting against generalized oxidative stress [14,15].

GPX3 is the only known extracellular glycosylated enzyme capable of utilizing thioredoxin, glutaredoxin, and glutathione as electron donors, which enables it to reduce a variety of hydroperoxides [16]. GPx3 is predominantly synthesized in the proximal tubules of the kidney [17]. Its expression may be related to oxidative stress caused by lipocyte metabolism. Furthermore, GPX3 may have functions beyond detoxification, potentially regulating cell growth and proliferation [18,19].

The GPX family has been identified to be highly expressed in various human cancers, including RCC [10,15,20–28]. Recent literature indicates differing levels of GPX1 and GPX3 in human tumors, with distinct implications for cancer progression [29]. For instance, Min et al. have highlighted GPX1 deactivation through methylation in gastric cancer, where there’s a notable loss of GPX1 and GPX3 expression [30]. Conversely, Yagublu et al. [22] demonstrated overexpression of GPX1 and GPX4 in colorectal adenocarcinoma, while research by Murawaki et al. reported lower GPX1 expression in colorectal cancer tissues compared to normal tissues [31].

In RCC, GPX1 overexpression may serve as a diagnostic and prognostic biomarker [15,25], as elevated GPX1 levels have been associated with adverse outcomes, underscoring the complexity of GPX involvement in RCC prognosis [14]. In contrast, GPX3 is considered a tumor suppressor gene [12,29], and diminished GPX3 expression may indicate poor outcomes for RCC patients [16]. However, the biological roles of GPX1 and GPX3 in ccRCC remain uncertain.

In the current study, we hypothesized that GPX1 and GPX3 are differentially expressed in ccRCC compared to non-neoplastic renal parenchyma and that their expression levels may correlate with tumor aggressiveness. Consequently, we aimed to analyze the immunophenotypic expression of GPX1 and GPX3 in ccRCC across different histological grades to clarify their potential roles in characterizing ccRCC and tumor progression.

## 2 Materials and Methods

### 2.1 Patients

We randomly retrieved histopathological tissue samples from 32 surgical specimens of RCC from our archive at the Department of Pathology, University Hospital of Larissa, Thessaly, Greece [Institutional Review Board and Ethics Committee of Faculty of Medicine, School of Health Sciences, University of Thessaly, Larissa, Greece (3124/29-7-2016)]. These specimens were collected from patients who underwent total nephrectomy at the University General Hospital of Larissa (from 2001 to 2005). Among the patients, 20 (62.5%) were male, and 12 (37.5%) were female, with ages ranging from 34 to 86 years (mean 65.18 ± 13.34 years) (Table 1). The average tumor size was 7.04 ± 2.63 cm, with a minimum size of 4 cm and a maximum of 13 cm. All specimens had been previously fixed in an aqueous formaldehyde solution and embedded in paraffin (FFPE) for histological evaluation at the Department of Pathology, University Hospital of Larissa.

**Table 1:** Clinical characteristics of renal cell carcinoma (RCC) patients and histopathological findings of the analyzed tumors.

Characteristic	Category	Value
Number of patients with RCC	Total	32 (100.0%)
	Male	20 (62.5%)
	Female	12 (37.5%)
Age (years)	Mean (±SD)	65.18 (±13.29)
	Range	34–86
	≥60	22 (68.8%)
	<60	10 (31.2%)
Tumor location	Left kidney	21 (65.6%)
	Right kidney	11 (34.4%)
Tumor size (cm)	Mean (±SD)	7.04 (±2.63)
	Range	4–13
Capsule infiltration‡	Yes	12 (37.5%)
	No	20 (62.5%)
Renal vein infiltration	Yes	3 (9.4%)
	No	28 (90.6%)
Renal pelvis infiltration	Yes	9 (28.1%)
	No	23 (71.9%)
Infiltration of the perirenal fat†	Yes	10 (31.2%)
	No	22 (68.8%)
Peripelvic fat infiltration#	Yes	7 (21.8%)
	No	25 (78.2%)
Necrosis	Yes	15 (46.8%)
	No	17 (53.2%)
WHO/ISUP (nucleolar) grade	Grade 1	1 (3%)
	Grade 2	12 (37.5%)
	Grade 3	15 (47%)
	Grade 4	4 (12.5%)

Note: ‡The tumor grew through the outer fibrous layer (capsule); †Also referred to as peripheric fat invasion: fat surrounding the outside of the kidney (outside the renal capsule); #Invasion of the fat within the renal sinus (central area). SD, standard deviation.

### 2.2 Immunohistochemical Analysis

We analyzed tumor tissue sections from FFPE specimens from our cohort of ccRCC using immunohistochemical (IHC) techniques, following standard procedures as previously described [32,33]. We analyzed by IHC at least three sections per sample.

In brief, we used primary antibodies for GPX1 (1:50 of rabbit Polyclonal; AP51937PU-N, OriGene, Rockville, MD, USA) and GPX3 (1:200 of rabbit polyclonal; NBP1-06398, Novus Biologicals, Centennial, CO, USA). After incubating the tissue sections with the primary antibodies overnight at 4°C, we applied a polymer-peroxidase method using EnVision+/Horseradish peroxidase (HRP) from DAKO, Glostrup, Denmark. To visualize the bound antibodies, we used the chromogen substrate 3,3’-diaminobenzidine (DAB).

Subsequently, we counterstained the sections with hematoxylin and mounted them in DPX mounting medium (BDH Laboratory Supplies, London, UK). Non-neoplastic renal parenchyma from normal adjacent kidney tissue served as controls for the immunostaining of GPX1 or GPX3 (Supplementary Fig. S1). For the negative control, we incubated histological sections with non-antigenic serum instead of the primary antibody.

### 2.3 Evaluation of Immunohistochemical Staining

First, we evaluated the localization of immunohistochemical staining for each GPX protein. We quantified the extent of GPX immunostaining in the tumor tissue section as a percentage (%) of positivity. Specifically, staining was assessed using an optical microscope (Zeiss Axioscope; Carl Zeiss AG, Germany), where we recorded both the extent of staining in the positive tumor areas and the intensity of staining.

Absence or weak staining of sporadic cells was assigned a score of 0, while staining that was weak, moderate, or intense compared to positive controls was scored as 1, 2, and 3, respectively. The staining intensity scores (ranging from 0 to 3) were then multiplied by the percentage (%) of tumor or tissue area that exhibited positive staining to yield a total IHC expression score (immunoscore). Therefore, the staining scale ranged from a minimum value of 0 (indicating absence or weak staining of sporadic cells) to a maximum value of 300 (representing 100% of cells with a staining intensity score of 3) (Supplementary Table S1).

We defined an immunoscore of GPX1 or GPX3 of ≥180 as high and an immunoscore of <180 as low to moderate.

### 2.4 Statistical Analysis

We examined the association between the expressions of GPX1 and GPX3 proteins and various histopathological, clinical, and demographic characteristics. To analyze the data, we used Spearman’s Rank-Order or Point-Biserial Correlation [Statistical Package for the Social Sciences (SPSS 30.0)]. We grouped the variables, as follows: sex (0 = male, 1 = female), age (0: <60 years; 1: ≥60 years), renal tumor location (0 = left kidney, 1 = right kidney), and the presence of capsule, pelvis or vein infiltration, perirenal or peripelvic fat infiltration, and necrosis (0 = absent, 1 = present). These groupings were used as qualitative variables (Supplementary Table S1).

## 3 Results

### 3.1 Histological Findings

Histological evaluation revealed that ccRCC was present in the left kidney in 65.6% (21 out of 32) of the cases examined, whereas 34.4% (11/32) of the tumors were located in the right kidney (Table 1 and Supplementary Table S1). Infiltration into the capsule, vein, or pelvis was identified in 37.5%, 9.4%, and 28.1% of the cases examined, respectively. Additionally, perirenal or peripheric fat infiltration was detected in 31.2% of cases, while peripelvic fat infiltration was observed in 21.8% of cases. Necrosis was noted in 46.8% of kidneys (Table 1).

**Figure 1:**
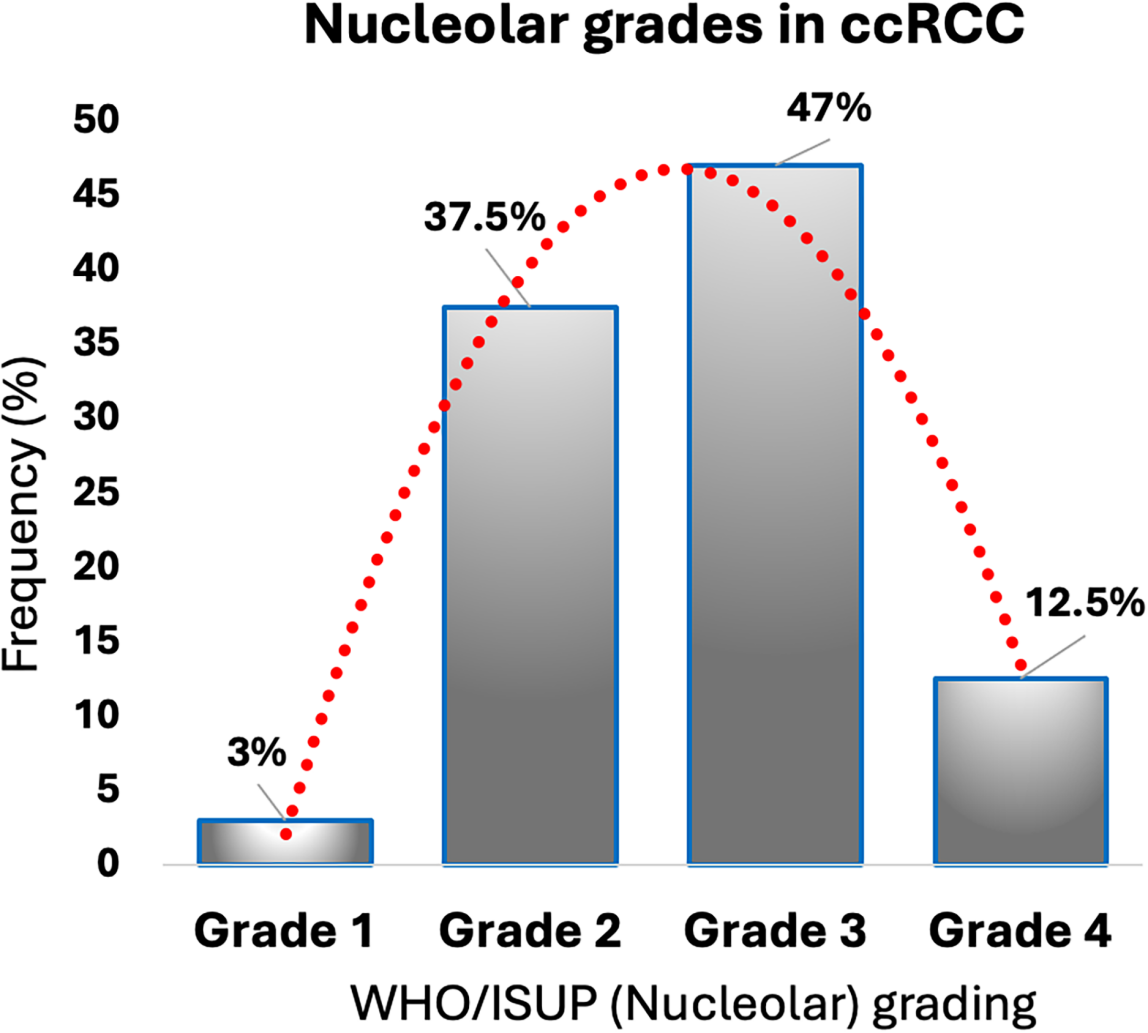
WHO/ISUP (nucleolar) grading distribution in clear cell renal cell carcinoma (ccRCC).

The nucleolar grade ranged from 1 to 4. Specifically, grades 1, 2, 3, and 4 were found in 3.1%, 37.5%, 46.9%, and 12.5% of ccRCC cases, respectively (Fig. 1; Table 1). Notably, almost 73.7% (14 out of 19) of advanced-grade tumors (grades 3–4) were located in the left kidney, while 46.2% (6 out of 13) of lower-grade tumors (grades 1–2) were found in the right kidney (Supplementary Table S1).

### 3.2 GPX1 Immunohistochemical Expression in ccRCC

**Figure 2:**
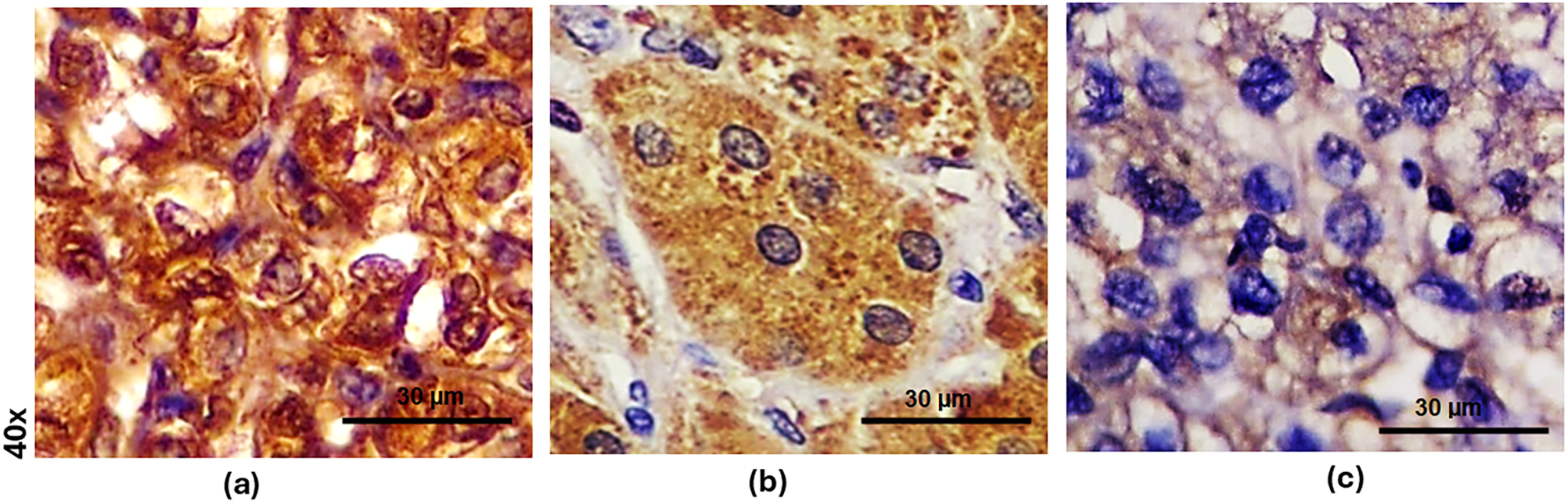
GPX1 immunohistochemical staining in clear cell renal cell carcinoma (ccRCC). **(a)** Strong (+3) cytoplasmic expression (immunoscore 300) in ccRCC with nucleolar grade 3. (**b**) Moderate (+2) cytoplasmic GPX1 expression (immunoscore 160) in ccRCC with nucleolar grade 2. (**c**) Weak (+1) focal GPX-1 expression (immunoscore 80) in ccRCC with nucleolar grade 3. Scale bars: 30 μm.

Microscopic examination showed that all analyzed ccRCC were positive for GPX1 immunostaining (100.0%; 32/32). As illustrated in Fig. 2, the GPX1 protein was localized in the cytoplasm of the tumor cells examined.

**Figure 3:**
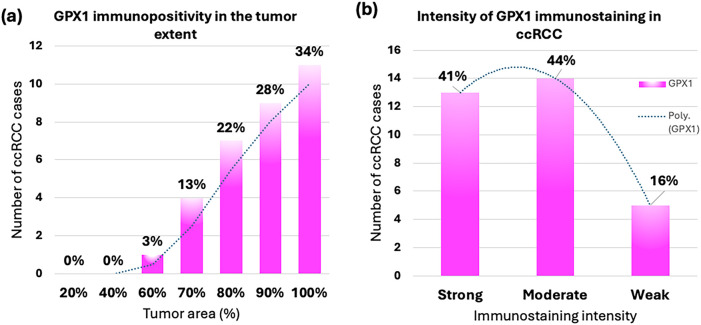

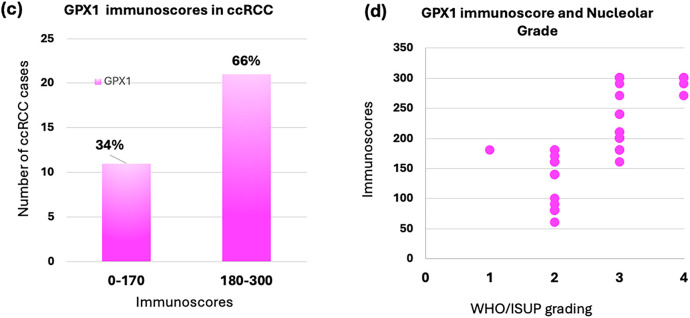
Immunohistochemical (IHC) analysis results for GPX1 in clear cell renal cell carcinoma (ccRCC). Distribution of ccRCC cases in relation to (**a**) GPX1 immunopositivity in the range of 0–100% of the tumor area; (**b**) weak, moderate, and strong intensity of GPX1 immunostaining; and (**c**) low to moderate (<180) or high (180–300) GPX1 immunoscores; (**d**) distribution of GPX1 immunoscore in relation to WHO/ISUP (nucleolar) grades of ccRCC.

The evaluation of GPX1 immunopositivity, including tumor extent, intensity of GPX1 immunostaining, and the calculated GPX1 immunoscore, along with its distribution relative to nucleolar grade, is presented graphically in Fig. 3.

Positive immunostaining for GPX1 was detected in a range of 60% to 100% of the tumor area in the studied cases. Notably, 84.4% of ccRCC samples (27 out of 32) exhibited high levels of GPX1 immunostaining, with 80% to 100% of the tumor area showing positive results. In contrast, only 15.6% (5 out of 32) displayed lower levels of GPX1 positivity, ranging from 60% to 70% (Fig. 3a).

The intensity of GPX1 immunostaining was recorded as strong to moderate in 84.4% of ccRCC samples. Specifically, strong (3+) or moderate (2+) intensities were found in 40.6% (13 out of 32) and 43.8% (14 out of 32) of the tumors, respectively. Weak (1+) intensity was observed in only 15.6% (5 out of 32) of the cases (Fig. 3b).

An immunoscore assessment was conducted based on the percentage of GPX1-positive neoplastic cells and the intensity of immunostaining, resulting in scores ranging from 60 to 300. As shown in Fig. 3c (Supplementary Table S1), a high GPX1 immunoscore between 180 and 300 was found in the majority of ccRCC cases (65.6%, or 21 out of 32), while a low to moderate GPX1 immunoscore (less than 180) was recorded in a smaller proportion (34.4%, or 11 out of 32).

Representative cases of ccRCC exhibiting strong, moderate, or weak GPX1 immunostaining intensities, along with high or low to moderate immunoscores, are displayed in Fig. 2a–c.

### 3.3 GPX3 Immunohistochemical Expression in ccRCC

**Figure 4:**
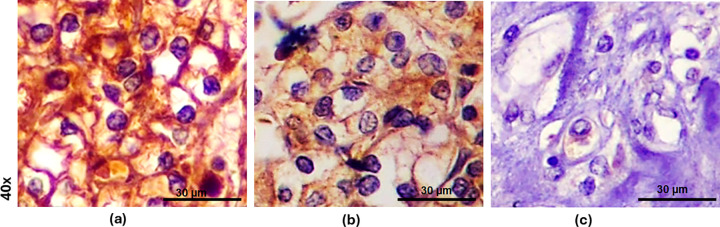
GPX3 immunohistochemical staining in clear cell renal cell carcinoma (ccRCC). (**a**) Strong (+3) cytoplasmic expression of GPX3 (immunoscore 300) in ccRCC with nucleolar grade 2. (**b**) Moderate (+2) focal expression of GPX3 (immunoscore 160) in ccRCC with nucleolar grade 3. (**c**) Negative (0) GPX3 immunostaining (immunoscore 0) in ccRCC. Scale bars: 30 μm.

Microscopic examination revealed that 96.9% of the analyzed ccRCC samples (31 out of 32) exhibited positive immunostaining for GPX3. As illustrated in Fig. 4, similar to GPX1, the GPX3 protein was localized in the cytoplasm of the tumor cells.

**Figure 5:**
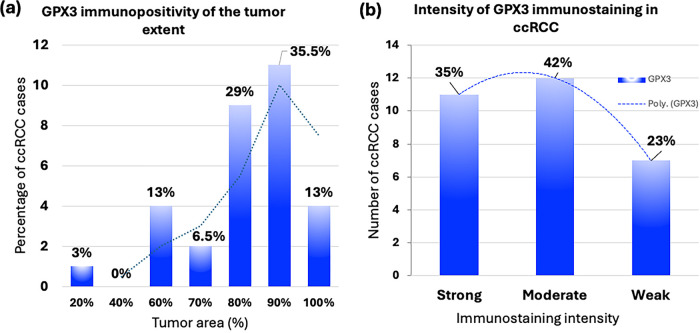

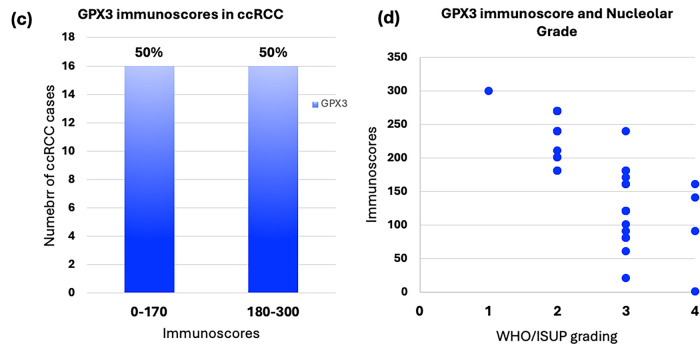
Immunohistochemical (IHC) analysis results for GPX3 in clear cell renal cell carcinoma (ccRCC). Distribution of ccRCC cases in relation to (**a**) GPX3 immunopositivity in the range of 0–100% of the tumor area; (**b**) weak, moderate, and strong GPX3 immunostaining intensity; and (**c**) low to moderate (<180) or high (180–300) GPX3 immunoscores; (**d**) distribution of GPX3 immunoscore in relation to WHO/ISUP (nucleolar) grade of ccRCC.

The evaluation of GPX3 immunopositivity, including tumor extent, intensity of immunostaining, and the calculated GPX3 immunoscore, along with its distribution relative to the nucleolar grading, is presented graphically in Fig. 5.

Positive immunostaining for GPX3 was observed in tumor areas ranging from 20% to 100%. Notably, 77.5% of ccRCC cases (24 out of 31) exhibited GPX3 positivity in a high percentage (80–100%) of the tumor area. In contrast, 22.5% of cases (7 out of 31) showed low GPX3 expression, affecting only 20–70% of the tumor area (Fig. 5a).

The intensity of GPX3 immunostaining varied from strong to moderate in 77.4% of ccRCC cases (24 out of 31). Specifically, strong (3+) intensity was detected in 35.5% (11 out of 31) of the tumors, while moderate (2+) intensity was found in 41.9% (13 out of 31). A weak (1+) intensity was observed in nearly a quarter (22.6%; 7 out of 31) of the cases (Fig. 5b).

The immunoscore assessment of GPX3 immunostaining intensity ranged from 0 to 300. As shown in Figs. 5c, a low GPX3 immunoscore of less than 180 was found in half of the ccRCC cases (50.0%; 16 out of 32) (Supplementary Table S1).

Representative ccRCC cases with variations in GPX3 immunostaining intensity, including strong, moderate, or weak, as well as high or low to moderate immunoscores, are presented in Fig. 4a,b. Negative GPX3 immunostaining was noted in one case (Fig. 4c).

### 3.4 Correlation of GPX1 and GPX3 Protein Expression with the Histopathological Features of RCC

The statistical analysis revealed a significant positive correlation between the GPX1 immunoscore and nucleolar grade (*r* = 0.80, *p* < 0.0001). Additionally, a positive correlation was found between a high GPX1 immunoscore and several factors: renal vein infiltration (*r* = 0.37, *p* < 0.038), perirenal (*r* = 0.36, *p* < 0.043), and peripelvic fat infiltration (*r* = 0.43, *p* < 0.015). However, no statistically significant associations were found between GPX1 immunoscore and capsular infiltration, renal pelvis infiltration, or necrosis.

Furthermore, no significant correlations were found between the GPX1 immunoscore and age, sex, tumor location, or size (*p* > 0.05).

In contrast, the statistical analysis indicated a significant inverse correlation between the GPX3 immunoscore and nucleolar grade (*r* = −0.77, *p* < 0.0001). No significant correlations were identified between high or low GPX3 immunoscore and age, sex, tumor location, or size (*p* > 0.05).

Figs. 3d and 5d illustrate the distribution of GPX1 and GPX3 immunoscore in relation to nucleolar grade. Additionally, the GPX1 and GPX3 immunophenotypes for representative cases of ccRCC with varying nucleolar grades are presented in Figs. 2 and 4, respectively.

## 4 Discussion

RCC is the most common malignant tumor of the urogenital system. Approximately 25% of RCC patients have multiple metastatic findings in various organs, such as the liver, at the time of diagnosis. Previous studies have noted a correlation between left-sided RCC tumors and a higher risk of advanced-stage tumors, which aligns with our findings. However, for certain patient subgroups, tumor laterality may not be a significant predictor, as other factors, like tumor size, can influence outcomes [34,35]. Early screening for high-risk patients is crucial for preventing or treating the tumor at an early stage. Thus, identifying biomarkers for RCC could greatly enhance prognosis and therapeutic planning [36]. The immunophenotypes of specific molecular factors may provide insights into the initiation and progression of cancer [37–40]. In this study, we present data from the immunophenotypes of GPX1 and GPX3 in ccRCC to explore their potential roles in this histopathological context. Our findings support the notion that both GPX1 and GPX3 serve as surrogate histopathological markers in ccRCC and are linked to tumor aggressiveness.

Based on our data, GPX1 protein is highly expressed in ccRCC, and this expression is independent of patient age and gender. Our findings do not indicate any statistically significant correlations between GPX1 immunophenotypes and tumor size or subcellular localization, which aligns with previous studies [15]. In the ccRCC samples included in this study, GPX1 protein was localized to the cytoplasm of neoplastic cells. Cytoplasmic GPX1 was previously reported in RCC [15].

Using a semiquantitative method to calculate immunohistochemical expression in histological sections, our novel data indicate a significant correlation between high GPX1 immunoscore and high nucleolar grade. Given the prognostic significance of nucleolar grade, our results emphasize the complementary role of GPX1 immunophenotypes in assessing the aggressiveness of ccRCC. Furthermore, our data supports a previous report indicating elevated GPX1 levels in individuals diagnosed with ccRCC, which have been associated with advanced tumor grades and poor overall survival [25].

Additionally, we present a significant correlation between GPX1 protein expression and other adverse prognostic histological markers, such as renal vein infiltration, vascular infiltration, and perineural tumor infiltration, consistent with prior observations [10,15]. Notably, the study by Cheng et al. showed through IHC that the expression of GPX1 was significantly increased in the neoplastic cells of ccRCC compared to the adjacent non-neoplastic renal parenchyma [15]. This study also found that high GPX1 expression correlates with the stage of ccRCC [15]. Similarly, research by Wei et al., which analyzed the expression of GPX1 using the Oncomine database and Gene Expression Profiling Interactive, indicated that high GPX1 expression is associated with disease progression in patients with RCC [10].

Previous studies have discussed signaling pathways associated with RCC, such as hypoxic signaling [41]. This signaling regulates genes related to tumor progression, including angiogenesis, invasion, and metastasis. Other key pathways associated with RCC also include the PI3K/AKT/mTOR and wnt/β-catenin pathways [42]. The latter is particularly crucial for cell migration and may show varying expression levels in primary compared to metastatic ccRCC tumors [42,43]. Hypoxic signaling is mediated by hypoxia-inducible factors (HIFs), like HIF-1α, which can bind to GPX1 to promote its expression. Recent preclinical experiments have demonstrated that the up-regulation of GPX1, but not GPX3, contributes to hypoxia-induced GPX enzyme activity through HIF-1α activation [44]. Based on our data, we may speculate that a similar hypoxia-related molecular mechanism may mediate the increased protein expression of GPX1, potentially leading to the tumorigenesis and progression of ccRCC.

We also investigated immunophenotypes of GPX3 in all ccRCC included in our study. Our observations revealed that the GPX3 protein was localized in the cytoplasm of the neoplastic cells in 97% of cases. This cytoplasmic localization of GPX3 has been previously reported [16]. An interesting finding of our study, based on the semiquantitative analysis of the immunohistochemical expression, is that, unlike GPX1, higher expression levels of GPX3 correlate with lower nucleolar differentiation (grades 1 and 2) while GPX3 protein expression tends to be reduced in high-grade ccRCC. Moreover, GPX3 staining was found to be moderately positive in the epithelial cells of the adjacent non-neoplastic renal parenchyma (Supplementary Fig. S1). Although the number of carcinomas evaluated in our study is relatively limited, these results support the idea that reduced GPX3 protein levels in neoplastic renal cells may serve as a marker of tumor progression, consistent with data from earlier research. Specifically, Liu et al. demonstrated weak IHC expression of GPX3 in 54 cases of ccRCC, alongside intense staining in adjacent non-neoplastic renal tissue, suggesting that GPX3 may have a tumor suppressor role [16].

The reduced expression of GPX3 may be linked to various stages of cancer development and progression, including tumor initiation, invasion, and metastasis across different types of tumors [18,19]. However, the underlying mechanism is unclear. Generally, as we mentioned above, GPX3 functions as a tumor suppressor by inhibiting cancer cell growth, migration, and invasion through pathways such as NF-*κ*B and Wnt/β-catenin [45,46]. Both the NF-*κ*B and Wnt signaling pathways are crucial for the development and tissue homeostasis, and can be activated under hypoxic conditions, which is linked to HIF-2 [47]. Previous research has indicated that GPX3 can regulate HIF-2 levels [48]. Moreover, GPX3 promoter hypermethylation has been proposed as an epigenetic mechanism of its downregulation in ccRCC. This downregulation potentially leads to decreased GPX3 protein levels, as demonstrated in our findings. Recent research has also highlighted the role of post-transcriptional N6-methyladenosine (m^6^A) modification, mediated by methyltransferase-like 14 (METTL14), in suppressing the expression of GPXs in ccRCC [49]. Based on this, we can hypothesize that under hypoxic conditions, the overexpression of HIF-2 cannot be effectively suppressed, due to the reduced levels of GPX3 resulting from hypermethylation or post-translational modification. This, in turn, could activate oncogenic signaling pathways, contributing to the tumorigenesis and progression of ccRCC.

Inhibition of GPX3 expression, and consequently, its protein activity, may be linked to reduced apoptosis in cancer cells, as observed in prostate carcinomas [50]. Some researchers argue that GPX3 protects neoplastic cells from oxidative stress, which may subsequently worsen the overall survival of patients with ovarian carcinoma [51]. Analysis of GPX3 expression in a large cohort of ccRCC in relation to clinical parameters, treatment responses, and overall survival could yield valuable and potentially clinically relevant conclusions.

In the present study, we found no statistically significant correlations between GPX3 immunohistochemical expression and factors such as gender, age, tumor location, or tumor size. Additionally, we did not observe any correlation between GPX3 expression and renal vein infiltration, vascular infiltration, or perineural tumor infiltration. Similar to findings from previous studies by Liu et al. [16], GPX3 methylation was significantly associated with a higher nucleolar grade of RCCs; however, no significant correlations were found between methylation and gender, age, tumor location, TNM stage, or histological type.

## 5 Limitations

This study advances the understanding of oxidative stress-related enzymes in kidney cancer and provides a clinically relevant foundation for evaluating GPX1 and GPX3 as candidate prognostic biomarkers. Nonetheless, several limitations should be considered. The small sample size and lack of external validation are significant drawbacks. Accordingly, larger, well-powered studies, ideally with longitudinal follow-up, are warranted to more precisely define the association between GPX1/GPX3 immunoscores and patient survival and to confirm their prognostic utility. Additional validation in independent cohorts and complementary analytic approaches at the transcriptional level would further reinforce these results and more precisely delineate their associations with clinicopathological variables.

## 6 Conclusions

In conclusion, the results of the present study indicate that GPX1 and GPX3 proteins are expressed in ccRCC with a differing distribution between low-grade and high-grade tumors. Increased expression of GPX1 may be associated with unfavorable prognostic histopathological features. Conversely, the absence of GPX3 protein expression appears to be associated with increased tumor aggressiveness, as indicated by a higher degree of nucleolar grade according to the WHO/ISUP system. While the exact roles of GPX1 and GPX3 in carcinogenesis and disease progression remain unclear, further studies are encouraged to validate the potential role of GPXs as prognostic tissue biomarkers in ccRCC.

## Supplementary Materials



## Data Availability

All data generated or analyzed during this study are included in this published article and are available from the corresponding author upon reasonable request.

## Supplementary Materials

The supplementary material is available online at https://www.techscience.com/doi/10.32604/or.2026.077195/s1.
