# Supplementary material for: Glutathione Peroxidases 1 and 3 Immunoscores in Clear Cell Renal Cell Carcinoma: New Insights from a Case-Series Study
Source: Oncol Res. 2026 Apr 22;34(5):16. doi: 10.32604/or.2026.077195 (PMC13126390; doi:10.32604/or.2026.077195)
Supplement: Supplementary file 1 [file OncolRes-34-77195-s001.docx]

**Supplementary Materials**

**Supplemental Table S1:** Histological characteristics and GPXs immunophenotypes in clear cell renal cell carcinoma (ccRCC).

| **No.** | **Sex/Age** | **Tumor location** | **Tumor size** | **Histology** | **RCI** | **PVI** | **PFI** | **PPF** | **PCI** | **TN** | **WHO/ISUP (nucleolar) grades** | **GPX1 (extend*intensity) score** | **GPX3 (extend *intensity) score** |
| --- | --- | --- | --- | --- | --- | --- | --- | --- | --- | --- | --- | --- | --- |
| 1 | M/57 | L | 4 | ccRCC | 0 | 0 | 0 | 0 | 0 | 0 | 1 | (90*2) 180 | (100*3) 300 |
| 2 | M/59 | R | 4.5 | ccRCC | 0 | 0 | 0 | 0 | 0 | 1 | 2 | (60*1) 60 | (80*3) 240 |
| 3 | M/80 | R | 4.5 | ccRCC | 0 | 0 | 0 | 0 | 0 | 1 | 2 | (80*1) 80 | (90*3) 270 |
| 4 | M/72 | L | 4 | ccRCC | 1 | 0 | 1 | 0 | 0 | 1 | 2 | (80*1) 80 | (90*3) 270 |
| 5 | M/68 | L | 7 | ccRCC | 1 | 0 | 0 | 0 | 0 | 1 | 2 | (90*1) 90 | (80*3) 240 |
| 6 | M/47 | L | 5 | ccRCC | 0 | 0 | 0 | 0 | 0 | 0 | 2 | (100*1) 100 | (70*3) 210 |
| 7 | F/76 | R | 4.5 | ccRCC | 0 | 0 | 0 | 0 | 0 | 1 | 2 | (70*2) 140 | (100*2) 200 |
| 8 | F/47 | R | 9.5 | ccRCC | 0 | 0 | 0 | 0 | 1 | 0 | 2 | (70*2) 140 | (90*3) 270 |
| 9 | F/64 | R | 7 | ccRCC | 0 | 0 | 0 | 0 | 0 | 1 | 2 | (80*2) 160 | (80*3) 240 |
| 10 | F/66 | L | 9.5 | ccRCC | 0 | 0 | 0 | 0 | 0 | 0 | 2 | (80*2) 160 | (90*2) 180 |
| 11 | F/86 | R | 9 | ccRCC | 0 | 0 | 0 | 0 | 0 | 0 | 2 | (85*2) 170 | (100*2) 200 |
| 12 | M/75 | L | 5.5 | ccRCC | 1 | 0 | 0 | 0 | 0 | 0 | 2 | (90*2) 180 | (90*2) 180 |
| 13 | M/78 | L | 8 | ccRCC | 0 | 0 | 0 | 0 | 1 | 0 | 2 | (90*2) 180 | (90*3) 270 |
| 14 | M/41 | L | 7 | ccRCC | 0 | 0 | 0 | 0 | 0 | 0 | 3 | (80*2) 160 | (80*3) 240 |
| 15 | F/56 | L | 5.5 | ccRCC | 0 | 0 | 0 | 0 | 0 | 1 | 3 | (90*2) 180 | (85*3) 170 |
| 16 | M/73 | L | 6.5 | ccRCC | 1 | 0 | 1 | 0 | 0 | 0 | 3 | (90*2) 180 | (80*1) 80 |
| 17 | F/75 | L | 4 | ccRCC | 0 | 0 | 0 | 0 | 0 | 0 | 3 | (100*2) 200 | (60*1) 60 |
| 18 | M/60 | R | 6 | ccRCC | 0 | 0 | 1 | 0 | 1 | 1 | 3 | (100*2) 200 | (90*1) 90 |
| 19 | F/73 | L | 9.5 | ccRCC | 1 | 0 | 0 | 1 | 0 | 1 | 3 | (100*2) 200 | (100*1) 100 |
| 20 | M/72 | L | 13 | ccRCC | 0 | 0 | 0 | 1 | 1 | 1 | 3 | (70*3) 210 | (80*2) 160 |
| 21 | F/65 | R | 5.5 | ccRCC | 0 | 0 | 1 | 1 | 0 | 0 | 3 | (70*3) 210 | (90*2) 180 |
| 22 | M/49 | L | 7 | ccRCC | 0 | 0 | 1 | 0 | 1 | 0 | 3 | (80*3) 240 | (80*1) 80 |
| 23 | M/79 | R | 10 | ccRCC | 1 | 1 | 0 | 0 | 0 | 1 | 3 | (80*3) 240 | (60*2) 120 |
| 24 | M/69 | L | 5.8 | ccRCC | 0 | 0 | 0 | 0 | 0 | 1 | 3 | (90*3) 270 | (60*2) 120 |
| 25 | F/61 | R | 9 | ccRCC | 1 | 0 | 0 | 0 | 0 | 1 | 3 | (95*3) 285 | (20*1) 20 |
| 26 | F/82 | L | 4.5 | ccRCC | 0 | 0 | 1 | 1 | 1 | 0 | 3 | (100*3) 300 | (80*2) 160 |
| 27 | M/49 | R | 4 | ccRCC | 0 | 1 | 0 | 0 | 0 | 1 | 3 | (100*3) 300 | (60*2) 120 |
| 28 | M/34 | L | 5.5 | ccRCC | 1 | 0 | 0 | 1 | 0 | 0 | 3 | (100*3) 300 | (90*2) 180 |
| 29 | M/78 | L | 12 | ccRCC | 1 | 0 | 1 | 1 | 1 | 1 | 4 | (90*3) 270 | (90*1) 90 |
| 30 | M/51 | L | 6.5 | ccRCC | 1 | 0 | 1 | 0 | 1 | 0 | 4 | (95*3) 285 | (80*2) 160 |
| 31 | M/61 | L | 9 | ccRCC | 1 | 0 | 1 | 0 | 0 | 0 | 4 | (100*3) 300 | (70*2) 140 |
| 32 | F/83 | L | 13 | ccRCC | 1 | 1 | 1 | 1 | 1 | 0 | 4 | (100*3) 300 | (0*0) 0 |

Note: 1 = Yes; 0 = No. RCI, capsule infiltration; RVI, renal vein infiltration; PFI, perirenal fat infiltration; PPF, peripelvic fat infiltration; PCI, renal pelvis infiltration; TN, necrosis.

**Supplementary Figure S1:** Non-neoplastic renal parenchyma (normal adjacent kidney tissue) for GPX1 and GPX3 immunostaining. (a) Strong GPX1 expression in the glomerular cells and the epithelial cells of the proximal urinary tubules (40×). (b) Moderate cytoplasmic positivity for GPX3 in the cells of the urinary tubules and absence of expression in the glomerulus (40×). Scale bars: 30 μm.
